# Nonlinear EEG Decoding Based on a Particle Filter Model

**DOI:** 10.1155/2014/159486

**Published:** 2014-05-15

**Authors:** Jinhua Zhang, Jiongjian Wei, Baozeng Wang, Jun Hong, Jing Wang

**Affiliations:** Xi'an Jiaotong University, Qujiang Campus, West Building No. 5, No. 99 YanXiang Road, YanTa District, Xi'an, Shaanxi 710045, China

## Abstract

While the world is stepping into the aging society, rehabilitation robots play a more and more important role in terms of both rehabilitation treatment and nursing of the patients with neurological diseases. Benefiting from the abundant contents of movement information, electroencephalography (EEG) has become a promising information source for rehabilitation robots control. Although the multiple linear regression model was used as the decoding model of EEG signals in some researches, it has been considered that it cannot reflect the nonlinear components of EEG signals. In order to overcome this shortcoming, we propose a nonlinear decoding model, the particle filter model. Two- and three-dimensional decoding experiments were performed to test the validity of this model. In decoding accuracy, the results are comparable to those of the multiple linear regression model and previous EEG studies. In addition, the particle filter model uses less training data and more frequency information than the multiple linear regression model, which shows the potential of nonlinear decoding models. Overall, the findings hold promise for the furtherance of EEG-based rehabilitation robots.

## 1. Introduction

With the growth of the aging population, treatment needs for nervous system diseases (e.g., spinal cord injury or stroke) have become bigger and bigger. As an advanced form of medical technology, rehabilitation robots have great potential in terms of both rehabilitation treatment and nursing. In recent years, the rehabilitation robot has become a research hotspot in the fields of brain science, biomedical and rehabilitation engineering, intelligent information processing, bionics, and so on. The control strategies of rehabilitation robots include force control, force field control, and bioelectrical signal (EMG, EEG, etc.) control. Brain signals are recorded by electrodes, reflecting the summation of the synchronous and rhythmic activity of neurons. Compared with EMG, brain signals contain more motion patterns and are available for more people; they are thus more suitable as an information source for rehabilitation robots. Brain signals can be obtained by invasive or noninvasive methods, though signals acquired by invasive methods have high signal-to-noise ratio and spatial resolution, and invasive method-based rehabilitation robot research has made great strides in animal experiments [1–4].

Due to the inherent risks of surgery and the gradual degradation of signal integrity, the invasive method is difficult to promote in clinical application. Although neuronal data acquired noninvasively from the scalp via electroencephalography (EEG) has comparatively low signal-to-noise ratio and spatial resolution, it is more suitable to be used in rehabilitation robots and in clinical applications because of its directness, its security, the simplicity of its acquisition equipment, its easy operation, its lower cost, and its fewer environmental restrictions.

In recent years, researchers have tried to read human mind from the EEG to determine the movement intent of people to achieve a noninvasive intelligent prosthetic control using, for example, the motor imagery-based rehabilitation system [5], the MindWalker of the Twente University [6], and the lower exoskeleton control system studied by the University of Houston [7]. Researchers, who study the EEG signals, concentrated more on pattern classification and feature extraction based on event-related potentials [8], visual evoked potential [9], and mental tasks [10].

In order to better control prostheses using EEG, researchers have been trying to get more movement information from the EEG signal besides pattern recognition or time-frequency analysis to identify the intent to achieve movement. For instance, Zhao et al. used the duration of a specific image to define the intensity of the task control commands, thus providing continuous control of an additional parameter [11]. Logar controlled the clamping force according to the phase demodulation method based on EEG [12]. The literature [13] analyzed the relationship between the actual movement velocity and the image. But, this movement information for intelligent prosthesis control is also far less.

Researchers have conducted studies on motion information extraction from EEG based on research on motion information extraction methods from an invasive signal (mainly decoding method includes linear filtering [14–16], Kalman filtering [17, 18], linear equation of state [19], and support vector machines [20]). In 2009, Bradberry et al. proposed a method to continuously decode hand position, velocity, and acceleration from 55-channel EEG signals and established a mapping model between EEG and motion information [14]. On this basis, in 2010, Bradberry continuously decoded 2D center-out movements, unconstrained 3D center-out movements, and 3D finger gestures, showing that EEG signals also contain rich motion information in the macroscale movement [15]. Lv et al. used a Kalman filter to predict the hand velocity in a “self-routed” movement based on features such as amplitude and power spectrum [21]. Antelis et al. established a mapping model between EEG and motion information by training the recorded trajectory from the initial point to the fixed point or any target point and the features of EEG in the spatial and time-frequency domain through the support vector machine (SVM) [22]. However, the researches above are limited to the decoding of specific limb trajectory. In 2011, Presacco et al. proposed a method that decoded unconstrained treadmill walking from EEG [16] and successfully obtained the linear and angular kinematics of the ankle, knee, and hip joints during walking.

During research on motion information extraction from EEG, most researchers have used a multiple linear regression decoding model based on a neural decoding method from invasive signals. Antelis et al. pointed out that the use of a linear regression model implies that the relevant EEG component has to be in the same frequency range as the signal to be decoded and suggested that a nonlinear model should be used to relate the limb kinematics to EEG temporal sequences [23]. Because EEG is a recording of electrical activity along the scalp, which comes from the neurons via the skull, the conductivity of the skull is nonlinear. The multiple linear regression model cannot reflect the nonlinear component of EEG.

Some nonlinear invasive neural decoding methods such as neural networks, support vector machines, and particle filter [24] for the movement decoding from EEG provide a reference. Particle filter is a technique for implementing a recursive Bayesian filter by Monte Carlo simulations. The key idea is to represent the required density function by set of random samples (particles) with associated weights. Particle filtering algorithm has been successfully applied in invasive neural decoding. For example, Wood et al. used particle filtering to recursively infer hand kinematics and attentional state conditioned on neural firing rates with a monkey [25]. Kelly and Lee decoded the V1 neuronal activity using particle filtering with Volterra kernels [26]. Gao et al. described the Bayesian decoding of hand motion from firing activity using a particle filter [27].

This paper proposes a nonlinear decoding model based on a particle filter. The multiple linear regression model and particle filter model are evaluated and compared using two- and three-dimensional hand motion decoding experiments.

## 2. Decoding Methods

### 2.1. Decoding Model by Multiple Linear Regression

The multiple linear regression model assumes that the kinematics of the hand are related to the EEG signals at present and the EEG signals at the previous moment and assumes that the relationship between the EEG signals of each channel is linear. The corresponding weight of each EEG channel can be obtained through multiple linear regression, and the decoding model is given by

(1) $$\begin{array}{c} x\left(t\right)=a_{x}+\overset{N}{\underset{n=1}{\sum }}\;\overset{L}{\underset{k=0}{\sum }}b_{nkx}S_{n}\left(t-k\right),\\ y\left(t\right)=a_{y}+\overset{N}{\underset{n=1}{\sum }}\;\overset{L}{\underset{k=0}{\sum }}b_{nky}S_{n}\left(t-k\right),\\ z\left(t\right)=a_{z}+\overset{N}{\underset{n=1}{\sum }}\;\overset{L}{\underset{k=0}{\sum }}b_{nkz}S_{n}\left(t-k\right), \end{array}$$

where *x*(*t*), *y*(*t*), and *z*(*t*) are, respectively, the horizontal, vertical, and depth position of the hand at time sample *t*, *N* is the number of EEG channels, *L* is the number of time lags, *S*_*n*_(*t* − *k*) is the voltage measured at EEG channel *n* at time lag *k*, and the *a* and *b* variables are weights obtained through multiple linear regression.

### 2.2. Decoding Model by Particle Filter

We have implemented a particle filtering method for reconstructing hand movement information from EEG signals. And, in particle filter decoding (or Bayesian decoding), the object is to find, for each time *t*, the distribution of the unobserved signal *C*_*t*_ (where *C*_*t*_ = [*x*(*t*), *y*(*t*), *z*(*t*)] has been used to represent the position of hand at time step *t*, with two- or three-dimensional vector) that is the key position information, given observations *S*_1:*t*_, while the observation *S*_*t*_ represented the vector of EEG. Hence, we view the decoding problem as a statistical inference problem in which we could get a Bayesian estimate of the posterior *p*(*C*_*t*_ | *S*_1:*t*_) at every time step. Making certain independence and first-order Markov assumptions leads to a recursive estimate of the posterior:

(2) p(Ct ∣ S1:t)=1λp(St ∣ Ct) ×∫p(Ct ∣ Ct−1)p(Ct−1 ∣ S1:t−1)dCt−1,

where 1/*λ* is a normalizing constant.

Particle filter decoding consists of two statistical models: (1) a state motion model (or temporal prior),  *p*(*C*_*t*_ | *C*_*t*−1_), for a process *C*_*t*_ describing the evolution of the state we are trying to predict (here, position of hand) and (2) a measurement model (or an observation model, or likelihood), *p*(*S*_*t*_ | *C*_*t*_), specifying the probability distribution of the data *S*_*t*_ given the underlying state *C*_*t*_.

#### 2.2.1. The State Motion Model

The state motion model describes the distribution of the unobserved signal one step in the future, *C*_*t*+1_, given the current value of the signal *C*_*t*_. Here, we use a second-order model, and the state motion model is as follows:

(3) $$\begin{array}{c} C_{t+1}=AC_{t}+W, \end{array}$$

where *C*_*t*_ = [*x*(*t*), *y*(*t*), *z*(*t*)] is the key position information, *A* is the transfer matrix, and *W* is the Gaussian noise.

#### 2.2.2. The Measurement Model

The measurement model specifies the relationship between the unobserved signal *C*_*t*_ and the observation *S*_*t*_. We assume the conditional independence of the EEG channels where the likelihood for the EEG signals is taken to be a Gaussian distribution. So, with some other usual assumption [23], the measurement model can be expressed as

(4) p(St ∣ Ct)≅(2π)−m/2|∑|−1/2 ×exp⁡(−12(St−μ)T∑−1(St−μ)),

where the *m*-dimensional vector *μ* is the mean and the covariance is the positive definite matrix ∑. The main steps of the particle filter decoding model are as follows.

(1) Initialization. Produce the particle swarm {*c*_0_^*i*^}_*i*=1_^*N*_*s*_^ by the prior probability, and the weight of each particle is 1/*N*_*s*_.
(2) Update. Update the particle weights at time *k*:
  (5) $$\begin{array}{c} w_{k}^{i}=w_{k-1}^{i}p\left(s_{k}\;|\;c_{k}^{i}\right),\;i=1,2,\ldots ,N_{s}. \end{array}$$
  Then, normalize
  (6) $$\begin{array}{c} w_{k}^{i}=\frac{w_{k}^{i}}{\sum_{i=1}^{N_{s}}w_{k}^{i}}. \end{array}$$
  The least mean-square estimate of the unknown parameter *x* at time *k* can be obtained from
  (7) $$\begin{array}{c} {\hat{x}}_{k}\approx \overset{N_{s}}{\underset{i=1}{\sum }}w_{k}^{i}c_{k}^{i}. \end{array}$$
(3) Resampling. Get a new particle collection {*c*_0:*k*_^i*^, *i* = 0,1, 2,…, *N*_*s*_}.
(4) Prediction. Use the state equation to predict the unknown parameter *c*_*k*+1_^*i*^.
(5) At time *k* = *k* + 1, return to step (2).

We use one set of experimental data to train the particle filter model and another experimental data set to verify the model. The Pearson correlation coefficient (*r*) between the measured and reconstructed hand positions was computed.

## 3. Experimental Design

After giving informed consent, six healthy, right-handed subjects, aged 20–25 (4 men and 2 women) and with no history of neurological disease, participated in the experiments. The experiments included two- and three-dimensional hand motions. To verify the applicability of the two decoding methods, we first tested their validity in two-dimensional hand motion, and then we further tested their validity in three-dimensional hand motion.

### 3.1. Two-Dimensional Hand Motion Experiment

**Figure 1:**
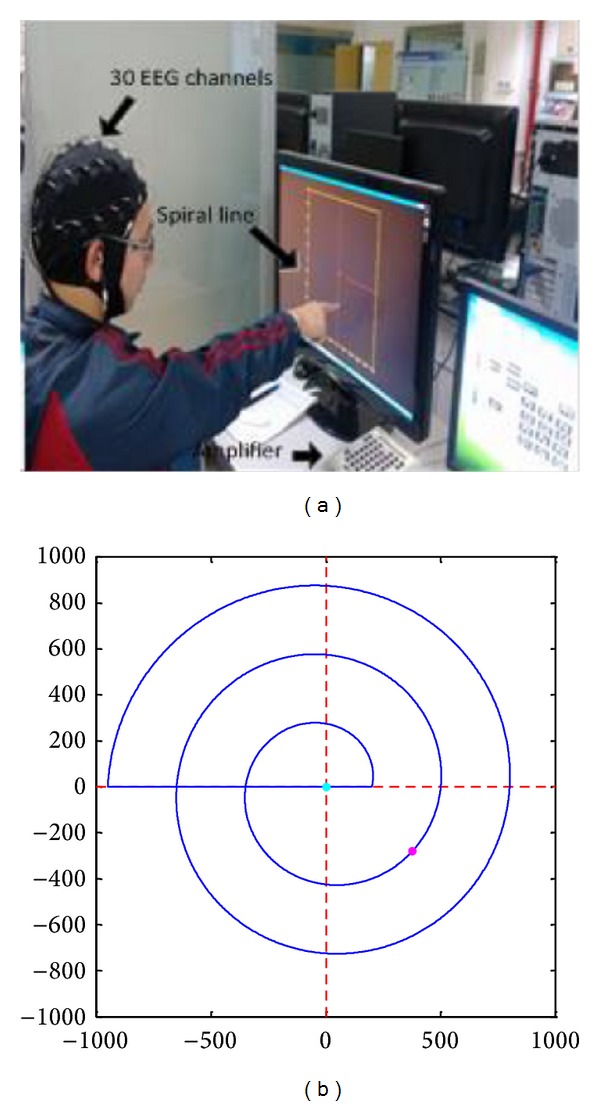
In the two-dimensional experiment, the participant moved his finger to track a computer-controlled cursor along the spiral line.

As shown in Figure 1, participants sat upright in a chair in front of the computer screen; the chair could be adjusted to obtain an appropriate height. During the experiment, participants were instructed to move their right arm/finger to track a computer-controlled cursor that moved along a spiral line in two dimensions on the computer screen. Meanwhile, EEG of participants was acquired. Participants were asked to keep other parts of their body except the right arm still and not to blink to reduce EMG and ocular artifacts. The most frontal electrodes (FP1, FP2) were removed offline from participants, as they are usually contaminated by eye blinks.

### 3.2. Three-Dimensional Hand Motion Experiment

**Figure 2:**
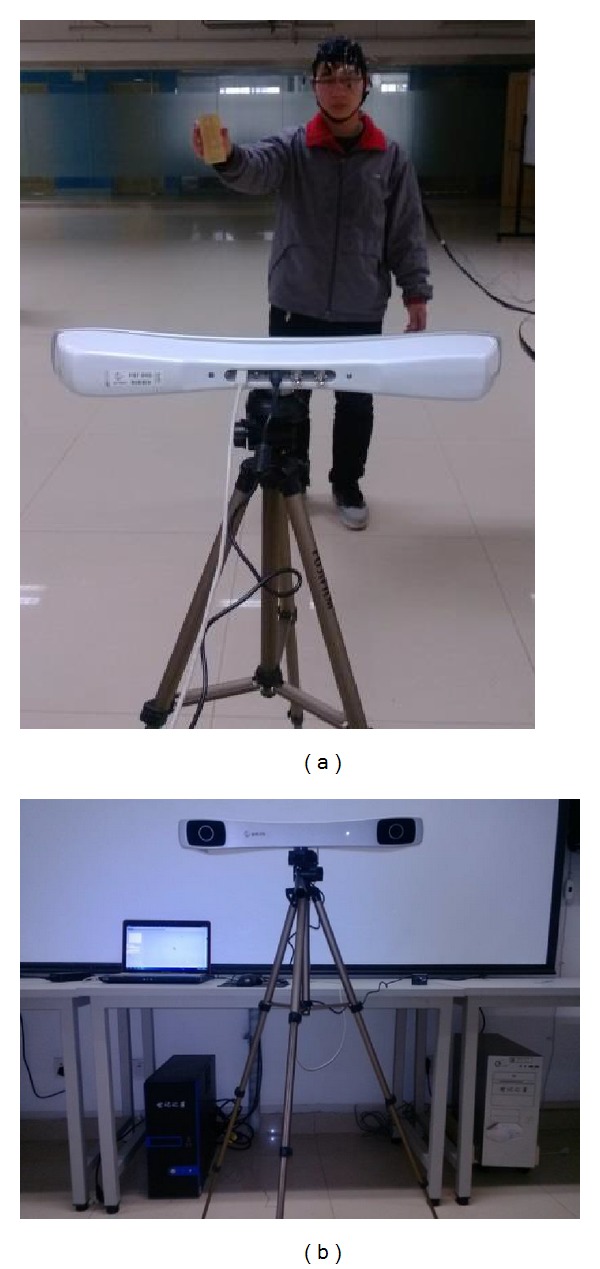
In the three-dimensional experiment, the participant swung his hand in space and his hand movement was tracked by the PST IRIS optical tracking system.

As Figure 2 shows, while wearing an EEG cap, participants held a target object whose three-dimensional motion was tracked by the optical tracking system. Participants swung their hands within a certain range in space slowly and freely. The body of participants remained still except for hand motions to minimize interference from EMG during the experiment, avoiding blinks to reduce ocular artifacts. The EEG signals from the FP1 and FP2 channels were also removed in the three-dimensional motion decoding. The optical tracking system was a PST IRIS motion capture device (PS-tech, Amsterdam, The Netherlands) with acquisition frequency of 120 Hz.

**Figure 3:**
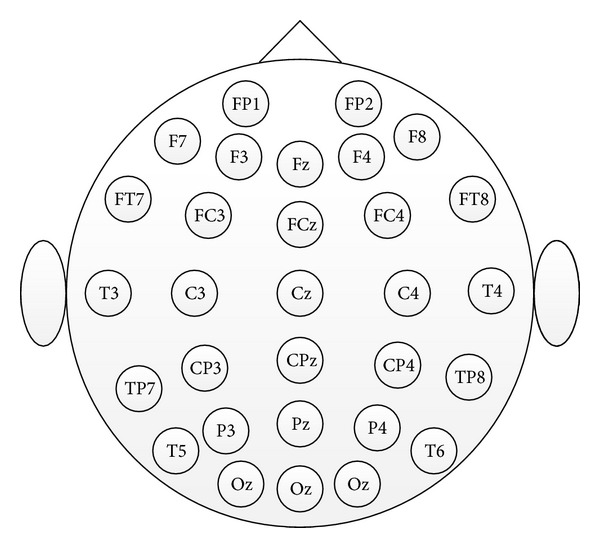
The location of 30 electrodes is an extended international 10–20 system.

A Neuroscan NuAmps Express system (Compumedics Ltd., VIC, Australia) was used to acquire EEG signals with the reference on the right mastoid process behind the right ear. The number of EEG channels, collection frequency, notch frequency, and low-pass cut-off frequency were 30, 500 Hz, 50 Hz, and 100 Hz, respectively. The location of 30 electrodes according to the extended international 10–20 system is shown in Figure 3.

In the two experiments, the hand movement and EEG signals were both acquired with timestamps. And they can be synchronized according to the timestamps.

### 3.3. Preprocessing

Vertical electroocular signals (VEOG) were measured with two electrodes attached superior and inferior to the orbital fossa of the left eye. And horizontal electroocular signals (HEOG) were measured with two electrodes attached to external canthi. In EEG signals recorded by a DC amplifier, baseline drift can occur artificially and simultaneously. First, the effects of baseline drift were removed. And the eye movements were removed from the EEG using a regression analysis. Then, the EEG signals were filtered with a 5th-order, low-pass Butterworth filter with a cut-off frequency of 2 Hz. The movement time of the small ball along the spiral line was 48 s in the two-dimensional experiment, and the number of coordinate positions of the small ball was 3079, so the ball movement sampling frequency (i.e., the sampling frequency of the hand motion) was about 64 Hz. Subsequently, the signals from each EEG channel were resampled from 500 Hz to 64 Hz, to ensure the same sampling frequency as that of the hand motion. The sampling frequency of the hand motion was 120 Hz in the three-dimensional experiment, so the EEG signals needed to be resampled from 500 Hz to 120 Hz. Then, the signals from each EEG channel were standardized according to the following:

(8) $$\begin{array}{c} \text{normalize}\left(\text{EEG}\right)=\frac{\left(\text{EEG}-\text{mean}\left(\text{EEG}\right)\right)}{\text{std}\left(\text{EEG}\right)}, \end{array}$$

where normalize(EEG)  is the normalized EEG signals, mean(EEG)  is the mean of EEG signals, and std(EEG)  is the standard deviation of EEG signals.

**Figure 4:**
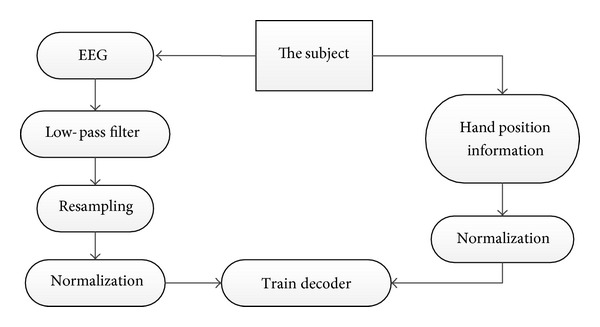
Experimental procedure. EEG and hand position information were recorded when subjects were conducting the experiment. After preprocessing, they were used to train and test the decoder.

The *X*, *Y*, and *Z* coordinates of the hand position during the hand movement were also standardized using the same equation. Finally, the hand position was decoded from EEG signals using the multiple linear regression and particle filter models. The entire process is shown in Figure 4.

## 4. Results

### 4.1. Multiple Linear Regression Model

**Figure 5:**
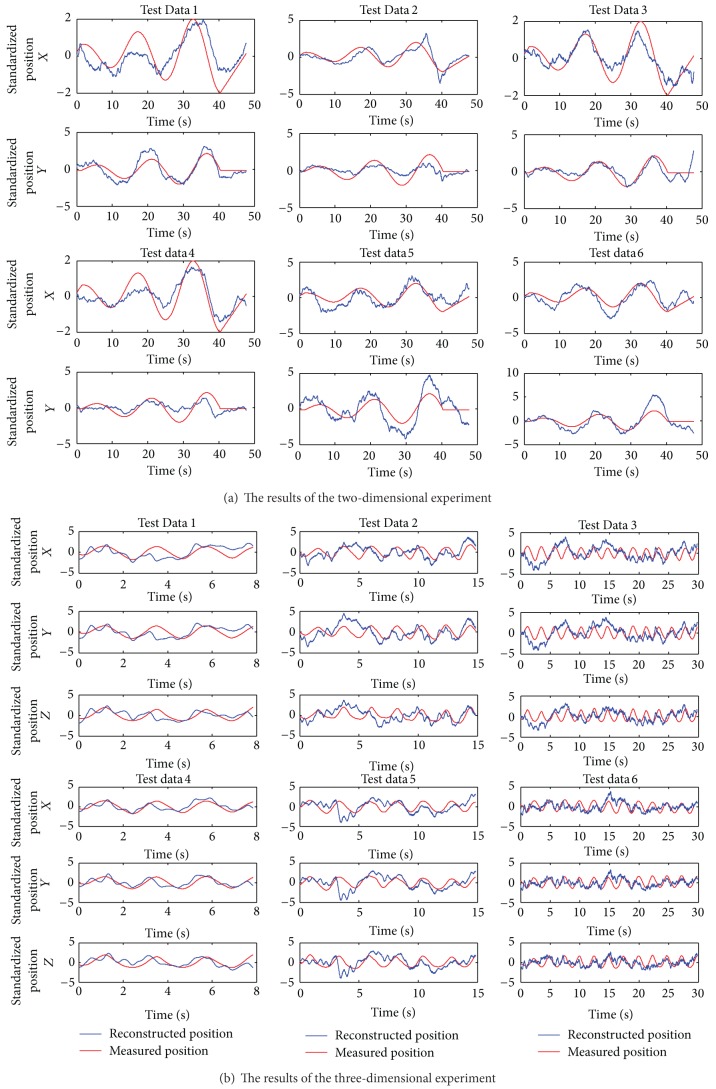
The measured and reconstructed hand position using the multiple linear regression model for Subject 1.

For each subject, data of 10 trials was collected under each experimental condition (2D, 3D (data length: 8 s, 15 s, and 30 s)). And 6 trials were selected from all the 10 trials according to the state of the subjects (concentration, movement of the body, etc.) during the trial. A 6 × 6-fold cross-validation procedure was employed to assess the reconstruction accuracy of the hand position from the EEG signals. In this procedure, the data of 5 trials was used for training; the remaining 1 trial data was used for testing. Figures 5(a) and 5(b) show, respectively, examples of the measured (red) and reconstructed (blue) hand positions in two and three dimensions in terms of decoding accuracy. The lengths of the data in the three-dimensional experiment include 8 s, 15 s, and 30 s. The 6 × 6-fold cross-validation procedure was used for every data length. In Figure 5(b), Test Data 1 and Test Data 4 are the results for data lengths of 8 s, Test Data 2 and Test Data 5 are the results for data lengths of 15 s, and Test Data 3 and Test Data 6 are the results for data length of 30 s.  Table 1 reports the Pearson correlation coefficients (*r*) in the two- and three-dimensional experiments for Subject 1.

### 4.2. Particle Filter Model

**Figure 6:**
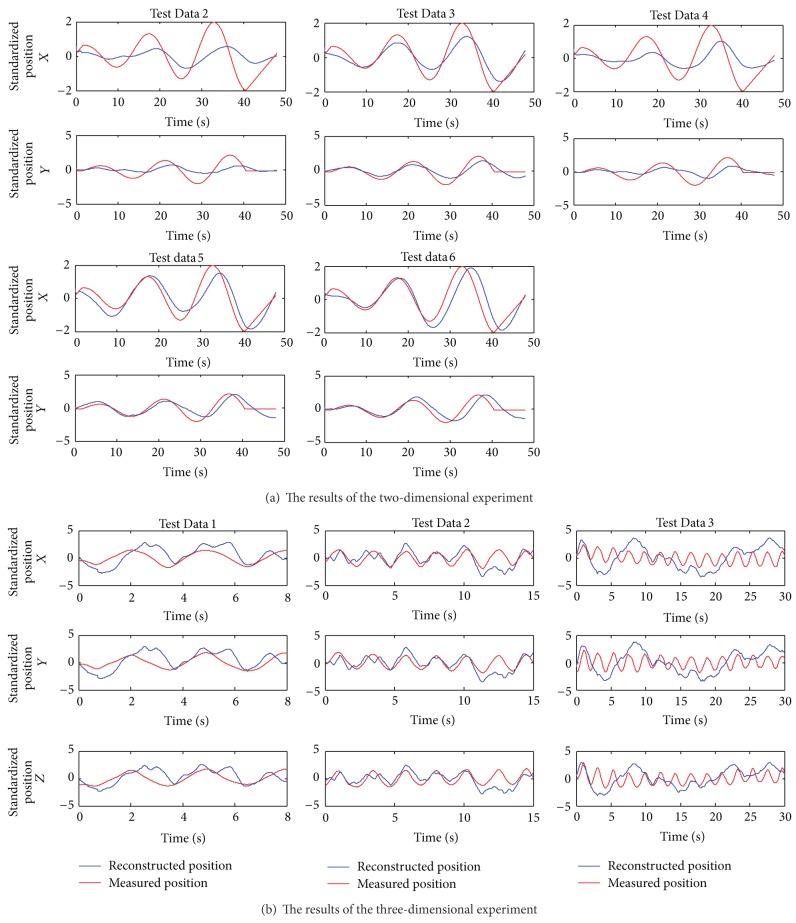
The measured and reconstructed hand positions using the particle filter model for Subject 1.

As a nonlinear decoding model, the particle filter model can reflect the nonlinear components of EEG signals and only needs one set of experimental data to train the model. For the two-dimensional experiment, we used Test Data 1 to train the particle filter model and the other five sets of experimental data to verify the model. The measured (red) and reconstructed (blue) hand positions in two dimensions are shown in Figure 6(a). For the three-dimensional experiment, we used the particle filter model for data of lengths 8 s, 15 s, and 30 s; the measured (red) and reconstructed (blue) hand positions in three dimensions are shown in Figure 6(b). The Pearson correlation coefficients (*r*) of Subject 1 in the two-dimensional and three-dimensional experiments are reported in Table 2.

**Figure 7:**
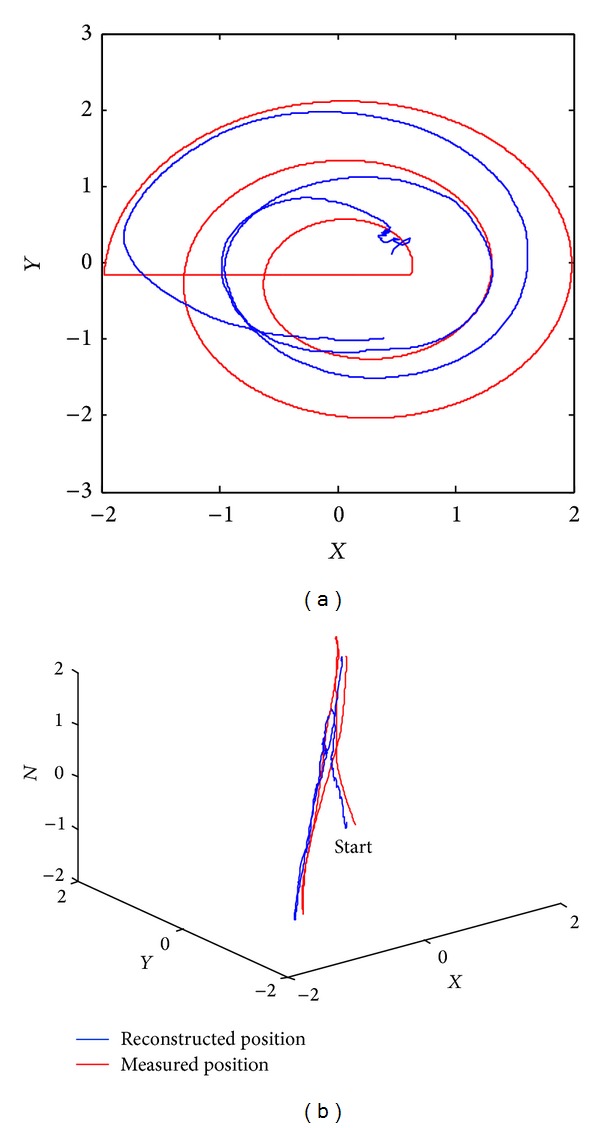
An example of hand movement trajectory of the subjects in 2- and 3-dimensional spaces. The word “start” in 3-dimensional space means the starting point.

The measured positions and decoded positions of hand movement in 2- and 3-dimensional spaces are shown in Figure 7. In order to avoid mess, we only selected a part of the hand trajectory in 3-dimensional space. As can be seen from Figure 7, the reconstructed position curve fits the measured position curve well.

With the multiple linear regression model, the mean and SD of the correlation coefficients across cross-validation procedure for all subjects are reported in Table 3, while the results of the particle filter model are shown in Table 4.

**Figure 8:**
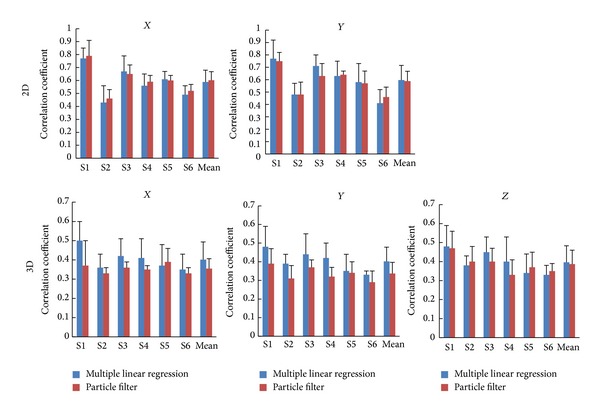
The mean value (*n* = 6) and standard deviation of correlation coefficients between measured and reconstructed hand positions for the six subjects (S1 through S6) using multiple linear regression model (blue) and particle filter model (red). The first row is for the two-dimensional experiment. The second row is for the three-dimensional experiment. The left column is for *X* direction, the middle column is for *Y* direction, and the right column is for *Z* direction.

In the two-dimensional experiment, multiple linear regression model and particle filter model have similar decoding accuracy (Figure 8). However, the three-dimensional decoding results of the multiple linear regression model are a little bit better than those of the particle filter model. The standard deviation of *r* from particle filter model is smaller, which means that it is more stable. It is evident that the decoding accuracy among subjects is variable. The results of Subject 1 are the best because he has conducted the experiments many times and became more adaptive.

**Figure 9:**
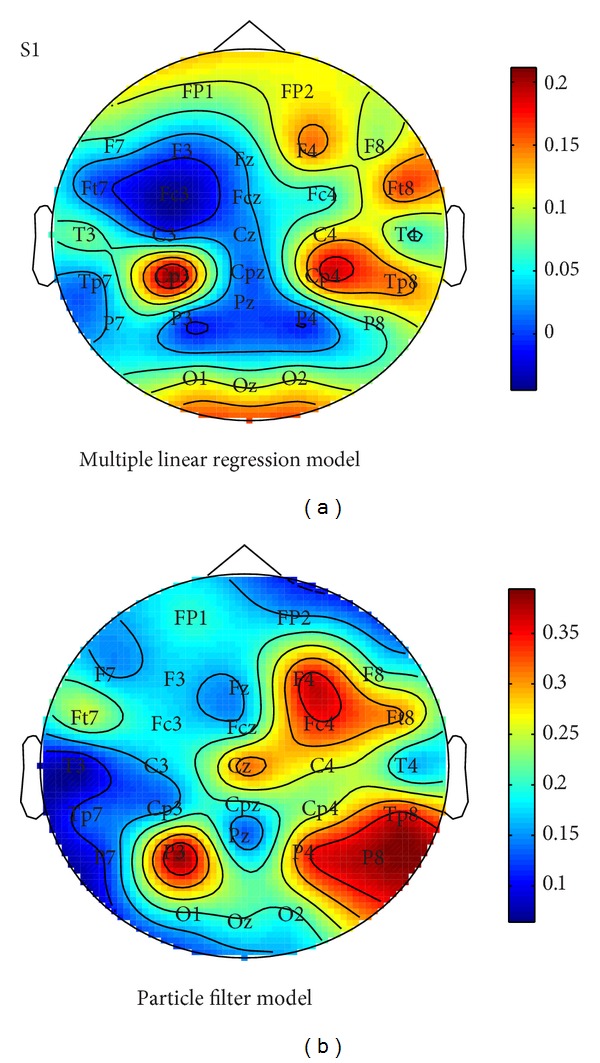
Spatial distribution of the contribution of each electrode for Subject 1 in multiple linear regression model (left) and particle filter model (right).

The topographies of the contribution of each electrode in the multiple linear regression model (at the best lag) and in the particle filter model were plotted in Figure 9. For the particle filter model, we used EEG signals of only one electrode to decode hand movement each time. And the correlation coefficient got from one electrode decoding was used to represent the contribution of this electrode. After the one electrode decoding procedure was used in all 30 electrodes, we can get the contribution of each electrode in the particle filter model. The topography of multiple linear regression model shows contributions from primary motor sensory area and occipital region. For the particle filter model, electrode locations at F4, Fc4, P3, P6, and TP8 are relevant for decoding right hand movement. It can be inferred that neural information about right hand movement is distributed across both hemispheres and the areas contributing to decoding are different in two decoding models.

## 5. Discussion

**Figure 10:**
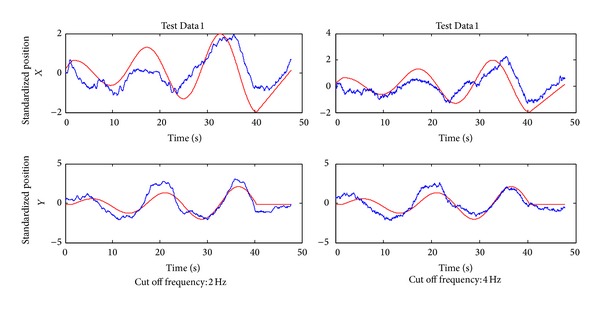
The decoding results using the multiple linear decoding model at different cut-off frequency of the low-pass filter.

As can be seen from Figure 5, the reconstructed curves in the *X* and *Y* directions in the two-dimensional experiment and the reconstructed curves in the *X*, *Y*, and *Z* directions in the three-dimensional experiment fit the measured curves well, showing the validity of the multiple linear regression model in the extraction of motion information from the EEG. However, there are high-frequency fluctuations in the reconstructed curves. One reason may be that the cut-off frequency of the low-pass filter during preprocessing is too high. As can be seen from Figure 10, the reconstructed curve with the cut-off frequency of 4 Hz has more frequent fluctuations than the one with the cut-off frequency of 2 Hz. It indicates that the cut-off frequency has an impact on the reconstructed curve. Reducing the cut-off frequency of the low-pass filter during preprocessing, or smoothing the reconstructed curve with a low-pass Butterworth filter, can effectively reduce these high-frequency fluctuations. Compared with the results in the two-dimensional experiment, the decoding accuracy in the three-dimensional experiment is low. The increased movement complexity may increase the difficulty of decoding.

In Figure 6(a), the reconstructed curves from the five sets of test data using the particle filter model fit the measured curves well in the trend. However, the decoding accuracies for Test Data 2 and Test Data 4 were relatively low, and the corresponding Pearson correlation coefficients are small. This may have been caused by inattention, eye blinks, or the movements of other parts of the body that introduced noise in the EEG data acquisition process for Test Data 2 and Test Data 4. The decoding accuracy for the three-dimensional experiment is smaller than that of the two-dimensional experiment, and Table 2 shows that the Pearson correlation coefficients for the data of lengths 8 s and 30 s in the three-dimensional experiment are smaller than that of the 15 s data. The same phenomenon exists when we analyze a large amount of data offline. Thus, the particle filter model may have an optimal data length. When the data length is shorter than the optimal data length, the number of iterations is small and leads to a low decoding accuracy. However, when the data length is longer than the optimal data length, the difference between the training data and test data becomes bigger, which also leads to a low decoding accuracy. Compared with the multiple linear regression model, the reconstructed curves from the particle filter model are smoother and have no high-frequency fluctuations. The Pearson correlation coefficients of the results—except Test Data 2 and Test Data 4 in the two-dimensional experiment—are above 0.7, showing a strong positive correlation. The reconstructed curves in the three-dimensional experiments, except Test Data 3, fit the measured curves well in the trend, showing the validity of the particle filter model.

**Figure 11:**
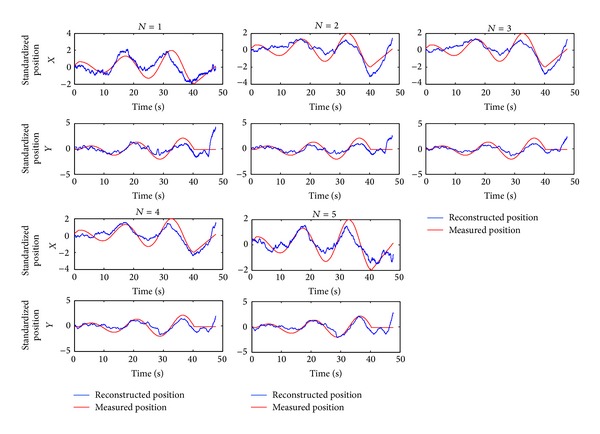
The influence of the number of training data sets on the multiple linear regression model.

**Figure 12:**
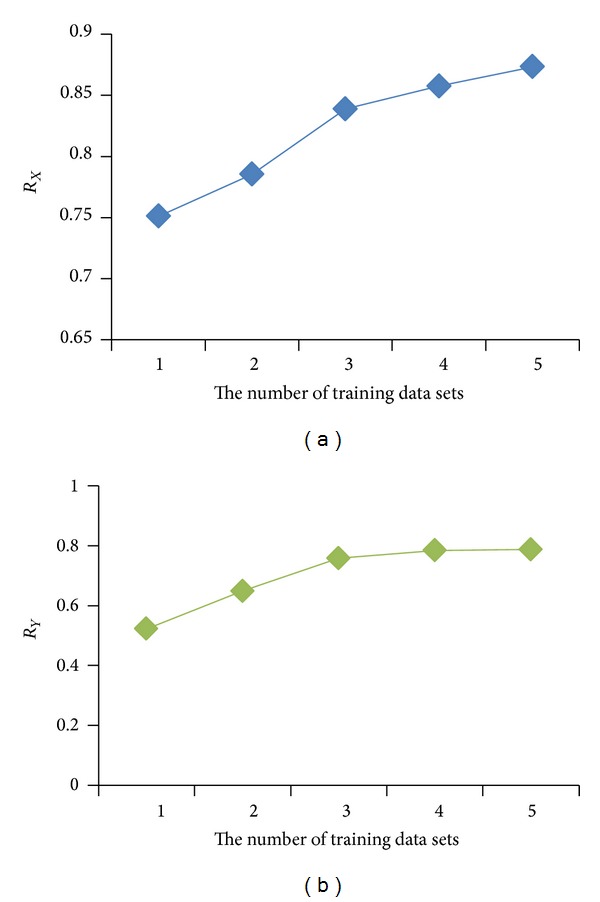
The changes in the Pearson correlation coefficient in the *X* direction (a) and the *Y* direction (b) with the number of training data sets.

Compared with the multiple linear regression model, the particle filter model uses less training data.  Figure 11 shows the decoding results using the multiple linear regression model when the number of the training data sets increases from 1 to 5.  Table 5 reports the corresponding Pearson correlation coefficients.  Figure 12 shows how the Pearson correlation coefficient changes with the number of training data sets in the *X* and *Y* directions, respectively.

As can be seen from Figure 12, the decoding accuracy of the multiple linear regression model in the *X* and *Y* directions increases as the number of training data sets increases. When the number of training data sets is small, the decoding accuracy increases rapidly with the increase in training data sets, and when the number of training data sets is large, the decoding accuracy increases slowly with the increase in training data sets. This is because when the number of training data sets is small, the influence of random interference factors, such as noise, in the training data is big. The influence of the random interference factors decreases with the increase of training data sets, as they can cancel each other out. As a result, a large training data set leads to a high decoding accuracy. When training data sets increase to a certain number, the impact of increasing the training data sets on the improvement of decoding accuracy becomes weaker. Thus, the decoding accuracy tends to stabilize. The decoding results in two dimensions in Table 2 show that the particle filter model can achieve high decoding accuracy using only one training data set. So when the number of experimental data is small, it is easier for the particle filter model to achieve high decoding accuracy.

**Figure 13:**
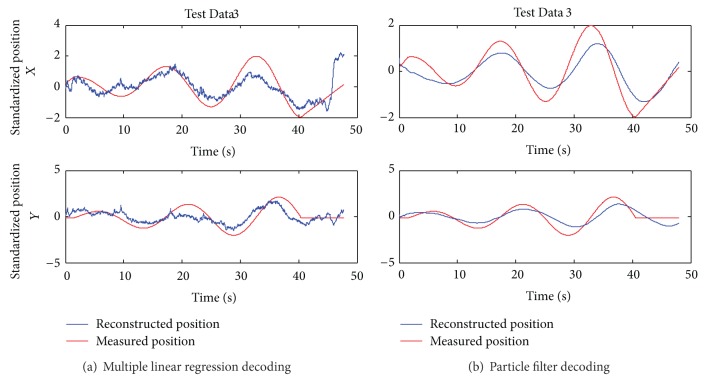
The two methods' processing capacities for high-frequency components of the EEG. The reconstructed positions from multiple linear regression model have fluctuations while the reconstructed positions from particle filter model are much smoother.

The high-frequency EEG components will cause high-frequency fluctuations in the reconstructed curves in the multiple linear regression model and lower its decoding accuracy. However, the particle filter model can process the high-frequency components of the EEG directly and use the information in a wide range of frequencies. Both the training data and test data are not low-pass filtered and are then used to decode the EEG using the multiple linear regression model and particle filter model. The decoding results are shown in Figure 13.

Figure 13 shows the two different methods' processing capacities for high-frequency components with the same EEG signal as an input. In Figure 13(a), for multiple linear regression, the Pearson correlation coefficients for the *X* and *Y* directions are 0.7228 and 0.6927, respectively, lower than the decoding accuracy of the results after low-pass filtering in Table 1, and have a bad denoising ability. Only when the EEG signal frequency is consistent with the hand-moving frequency can a high decoding accuracy be achieved. In experiments, the hand-moving frequency is below 2 Hz; hence, the EEG signals need to be processed by a low-pass filter with a cut-off frequency of 2 Hz. In Figure 13(b), for the particle filter decoding results, the Pearson correlation coefficients for the *X* and *Y* directions are 0.9044 and 0.8421, respectively, little different from the results after low-pass filtering in Table 2. Furthermore, it has a good effect on the processing of the high-frequency components. This is because the particle filter can process the high-frequency components of EEG in its algorithm, so the low-pass filter is not necessary. Moreover, the high-frequency components of the EEG may contain motion information; hence, the particle filter model can make use of more extensive frequency information, which may improve the decoding accuracy.

## 6. Conclusion

This paper used the multiple linear regression model and the particle filter model to decode the hand motion information in two and three dimensions from EEG signals and analyzed the decoding results and the factors that influenced it to compare the decoding features of the two methods and their usability. The experimental results showed that the multiple linear regression model needed multiple sets of training data to train and only worked well for low-frequency decoding. Conversely, for the particle filter model, only one set of training data was necessary, and the model could process EEGs containing high-frequency components, which means a more extensive utilization of frequency information. Nevertheless, the length of the EEG data affected the decoding accuracy. With this in mind, the decoding model should be carefully chosen in accordance with the model features and its application scenario.

Our future work is to research the influence of other factors on the decoding, optimize the particle filter method to improve its decoding accuracy, and try to perform online decoding and manipulator control.

## Figures and Tables

**Table 1:** The Pearson correlation coefficient (*r*) using the multiple linear regression model for Subject 1.

Two-dimensional	Test Data 1	Test Data 2	Test Data 3	Test Data 4	Test Data 5	Test Data 6
*R*_*X*_	0.6736	0.7875	0.8733	0.8586	0.6895	0.7408
*R*_*Y*_	0.8824	0.6723	0.7870	0.5195	0.8544	0.9019

Three-dimensional	Test Data 1	Test Data 2	Test Data 3	Test Data 4	Test Data 5	Test Data 6

*R*_*X*_	0.4214	0.4354	0.5385	0.6813	0.5339	0.4097
*R*_*Y*_	0.4558	0.3772	0.5237	0.6784	0.4995	0.3745
*R*_*Z*_	0.4656	0.3708	0.5028	0.6674	0.4918	0.3672

**Table 2:** The Pearson correlation coefficient (*r*) using the particle filter model for Subject 1.

Two-dimensional	Test Data 2	Test Data 3	Test Data 4	Test Data 5	Test Data 6
*R*_*X*_	0.6416	0.9248	0.7025	0.8658	0.8071
*R*_*Y*_	0.6884	0.8561	0.6761	0.7820	0.7532

Three-dimensional	Test Data 1	Test Data 2	Test Data 3		

*R*_*X*_	0.4512	0.5073	0.1390		
*R*_*Y*_	0.4635	0.5941	0.1569		
*R*_*Z*_	0.4687	0.3452	0.2849		

**Table 3:** Decoding accuracy using multiple linear regression model for all six subjects.

	Subject 1	Subject 2	Subject 3	Subject 4	Subject 5	Subject 6
Two-dimensional						
*R*_*X*_	0.77 ± 0.08	0.43 ± 0.13	0.67 ± 0.12	0.56 ± 0.09	0.61 ± 0.06	0.49 ± 0.07
*R*_*Y*_	0.77 ± 0.15	0.48 ± 0.09	0.71 ± 0.09	0.63 ± 0.12	0.58 ± 0.15	0.41 ± 0.11
Three-dimensional						
*R*_*X*_	0.5 ± 0.1	0.36 ± 0.07	0.42 ± 0.09	0.41 ± 0.1	0.37 ± 0.11	0.35 ± 0.08
*R*_*Y*_	0.48 ± 0.11	0.39 ± 0.05	0.44 ± 0.11	0.42 ± 0.08	0.35 ± 0.09	0.33 ± 0.02
*R*_*Z*_	0.48 ± 0.11	0.38 ± 0.05	0.45 ± 0.08	0.40 ± 0.13	0.34 ± 0.1	0.33 ± 0.05

**Table 4:** Decoding accuracy using particle filter model for all six subjects.

	Subject 1	Subject 2	Subject 3	Subject 4	Subject 5	Subject 6
Two-dimensional						
*R*_*X*_	0.79 ± 0.12	0.46 ± 0.07	0.65 ± 0.07	0.59 ± 0.05	0.6 ± 0.04	0.52 ± 0.05
*R*_*Y*_	0.75 ± 0.07	0.48 ± 0.1	0.63 ± 0.1	0.64 ± 0.03	0.57 ± 0.1	0.46 ± 0.08
Three-dimensional						
*R*_*X*_	0.37 ± 0.13	0.33 ± 0.03	0.36 ± 0.03	0.35 ± 0.02	0.39 ± 0.07	0.33 ± 0.03
*R*_*Y*_	0.39 ± 0.08	0.31 ± 0.07	0.37 ± 0.04	0.32 ± 0.05	0.34 ± 0.06	0.29 ± 0.06
*R*_*Z*_	0.47 ± 0.09	0.4 ± 0.08	0.40 ± 0.07	0.33 ± 0.08	0.37 ± 0.08	0.35 ± 0.04

**Table 5:** The Pearson correlation coefficients in the *X* and *Y* directions at different number of training data sets.

The number of data sets	*N* = 1	*N* = 2	*N* = 3	*N* = 4	*N* = 5
*R*_*X*_	0.751	0.7853	0.8389	0.8576	0.8733
*R*_*Y*_	0.5225	0.6488	0.7577	0.7838	0.787

## References

[1] O’Doherty JE, Lebedev MA, Ifft PJ (2011). Active tactile exploration using a brain-machine-brain interface. *Nature*.

[2] Clanton ST (2011). *Brain-computer interface control of an anthropomorphic robotic arm [Ph.D. thesis]*.

[3] Velliste M, Perel S, Spalding MC, Whitford AS, Schwartz AB (2008). Cortical control of a prosthetic arm for self-feeding. *Nature*.

[4] Carmena JM, Lebedev MA, Crist RE (2003). Learning to control a brain-machine interface for reaching and grasping by primates. *PLoS Biology*.

[5] Do AH, Wang PT, King CE, Chun SN, Nenadic Z (2013). Brain-computer interface controlled robotic gait orthosis: a case report. *Journal of NeuroEngineering and Rehabilitation*.

[6] https://mindwalker-project.eu.

[7] http://www.egr.uh.edu/news/201204/brain-controlled-exoskeleton-one-step-closer-reality.

[8] Picton TW, Bentin S, Berg P (2000). Guidelines for using human event-related potentials to study cognition: recording standards and publication criteria. *Psychophysiology*.

[9] Kim YJ, Grabowecky M, Paller KA, Muthu K, Suzuki S (2007). Attention induces synchronization-based response gain in steady-state visual evoked potentials. *Nature Neuroscience*.

[10] Liang N-Y, Saratchandran P, Huang G-B, Sundararajan N (2006). Classification of mental tasks from EEG signals using extreme learning machine. *International Journal of Neural Systems*.

[11] Zhao QB, Zhang QL, Andrzej C (2008). EEG based on asynchronous BCI car navigation system in 3D virtual reality environment. *Chinese Science Bulletin*.

[12] Logar V, Škrjanc I, Belič A (2008). Gripping-force identification using EEG and phase-demodulation approach. *Neuroscience Research*.

[13] Yuan H, Perdoni C, He B (2010). Relationship between speed and EEG activity during imagined and executed hand movements. *Journal of Neural Engineering*.

[14] Bradberry TJ, Gentili RJ, Contreras-Vidal JL Decoding three-dimensional hand kinematics from electroencephalographic signals.

[15] Bradberry TJ, Gentili RJ, Contreras-Vidal JL (2010). Reconstructing three-dimensional hand movements from noninvasive electroencephalographic signals. *Journal of Neuroscience*.

[16] Presacco A, Goodman R, Forrester L, Contreras-Vidal JL (2011). Neural decoding of treadmill walking from noninvasive electroencephalographic signals. *Journal of Neurophysiology*.

[17] Wu W, Shaikhouni A, Donoghue JP, Black MJ Closed-loop neural control of cursor motion using a kalman filter.

[18] Wu W, Black MJ, Mumford D, Gao Y, Bienenstock E, Donoghue JP (2004). Modeling and decoding motor cortical activity using a switching Kalman filter. *IEEE Transactions on Biomedical Engineering*.

[19] Wu W, Kulkarni JE, Hatsopoulos NG, Paninski L (2009). Neural decoding of hand motion using a linear state-space model with hidden states. *IEEE Transactions on Neural Systems and Rehabilitation Engineering*.

[20] Shpigelman L, Singer Y, Paz R, Vaadia E (2005). Spikernels: predicting arm movements by embedding population spike rate patterns in inner-product spaces. *Neural Computation*.

[21] Lv J, Li Y, Gu Z (2010). Decoding hand movement velocity from electroencephalogram signals during a drawing task. *BioMedical Engineering Online*.

[22] Antelis JM, Montesano L, Minguez J (2011). Towards decoding 3D finger trajectories from EEG. *International Journal of Bioelectromagnetism*.

[23] Antelis JM, Montesano L, Ramos-Murguialday A, Birbaumer N, Minguez J (2013). On the usage of linear regression models to reconstruct limb kinematics from low frequency EEG signals. *PloS ONE*.

[24] Brockwell AE, Rojas AL, Kass RE (2004). Recursive Bayesian decoding of motor cortical signals by particle filtering. *Journal of Neurophysiology*.

[25] Wood F, Prabhat P, Donoghue JP, Black MJ Inferring attentional state and kinematics from motor cortical firing rates.

[26] Kelly RC, Lee TS Decoding V1 neuronal activity using particle filtering with volterra kernels.

[27] Gao Y, Black MJ, Bienenstock E, Shoham S, Donoghuex JP (2002). Probabilistic inference of hand motion from neural activity in motor cortex. *Advances in Neural Information Processing Systems*.

